# Trends in Incidence and Mortality of Stroke in China From 1990 to 2019

**DOI:** 10.3389/fneur.2021.759221

**Published:** 2021-11-22

**Authors:** Tong Sun, Siyang Chen, Ke Wu, Min Sun, Xianyan Zhang, Chao You

**Affiliations:** ^1^Department of Neurosurgery, West China Hospital, Sichuan University, Chengdu, China; ^2^Health Management Center, West China School of Public Health and West China Fourth Hospital, Sichuan University, Chengdu, China; ^3^Department of Neurosurgery, Xichang People's Hospital, Liangshan, China

**Keywords:** burden, stroke, incidence, mortality, trends

## Abstract

**Objective:** Stroke is a leading cause of mortality and morbidity globally. This study aimed to analyze the burden and 30-year trends of ischemic stroke, intracerebral hemorrhage (ICH), and subarachnoid hemorrhage (SAH) in China.

**Methods:** Data that include incidence and mortality of stroke in China from January 1, 1990 to December 31, 2019 were derived from the Global Burden of Disease (GBD) study 2019. The absolute numbers of incident cases and deaths over the time, and age-standardized rates per 100,000, such as age-standardized incidence rate (ASIR) and age-standardized mortality rate (ASMR), were analyzed.

**Results:** In 2019, there were 3.9 (95% uncertainty intervals (*UI*) 3.4–4.5) million incident cases and 2.1 (3.4–4.5) million deaths related to stroke in China. The ASIR and ASMR of stroke in China was 200 (176–230) and 127 (110–144). From 1990 to 2019, the ASIR of ischemic stroke had increased by 35.0% (29.0–40.0) while the ASIR of ICH and SAH had decreased by −53.0% (−56.0 to −50.0) and by −39.0% (−44.0 to −35.0), respectively. The ASMR of ischemic stroke had increased by 3.0% (−26.0 to 16.0) while the ASMR of ICH and SAH had decreased by −48.0% (−59.0 to −38.0) and by −84.0% (−89.0 to −69.0), respectively.

**Conclusion:** Although the incidence and mortality rates of stroke in China were decreased from 1990 to 2019, the number of incident cases and deaths nearly doubled. A sharp increase in the incidence rate of ischemic stroke was observed. A higher incidence rate of ischemic stroke in the women was also observed.

## Introduction

Stroke is a predominant cause of mortality and morbidity globally (1). In the past few decades, the incident cases of stroke continued to increase leading to a high disease burden, particularly in the developing and low-income countries (2, 3). The results from the previous Global Burden of Diseases (GBD) study suggested that the age-standardized incidence and mortality rates decreased but the overall burden of stroke remained high (4, 5). China, as the biggest developing countries worldwide, had the largest number of incident cases and deaths related to stroke (6). In this light, a comprehensive analysis of the stroke-related burden in Chi]na, will provide evidence to the policymakers and healthcare workers, and implement the effective prevention strategies (7).

In the current study, we aimed to provide the first systematic analysis of the trends in the incidence and mortality of the strokes, such as ischemic stroke, intracerebral hemorrhage (ICH), and subarachnoid hemorrhage (SAH) in China based on the most recent GBD study 2019. We analyzed the absolute numbers of the incident cases and deaths over the time, and age-standardized rates per 100,000, such as age-standardized incidence rate (ASIR) and age-standardized mortality rate (ASMR), which were compared across the different age groups and sex.

## Methods

Data, such as incidence and mortality in 204 countries and regions from 1990 to 2019, were collected through the GBD Results Tool (http://ghdx.healthdata.org/gbd-results-tool) on the website of Institute of Health Metrics and Evaluation (IHME). The detailed methods utilized to generate estimates were described previously (8–11). The GBD study 2019 was conducted from January 1, 1990 to December 31, 2019 and the data analysis was completed on October 1, 2020. The overall incidence of stroke was estimated utilizing a Bayesian meta-regression model (DisMod-MR 2.1) and the standard Cause of Death Ensemble modeling (CODEm) method was utilized to determine the estimates of mortality (12). Each estimate was calculated from the mean of 1,000 draws from the posterior distribution by age, sex, location, and year. The 95% uncertainty intervals (UIs) were the 25th and 975th values of the ordered draws. A 95% *UI* excluding zero for all the estimates was defined as statistically significant. The data were described as the estimates (95% *UI*).

In this study, ischemic stroke was considered if an atherosclerotic and thromboembolic event occurred leading to insufficient blood flow to brain and cerebral infarction while ICH and SAH were defined as a non-traumatic, primary event identified by brain imaging (5). An incident case of stroke was defined as the occurrence of first-ever stroke based on the WHO diagnosis criteria (13). Specifically, a transient ischemic attack (TIA) was excluded in the GBD study. The figures were generated through the GBD Results Tool, as well as the GBD Compare Tool.

## Results

### Incidence

**Figure 1:**
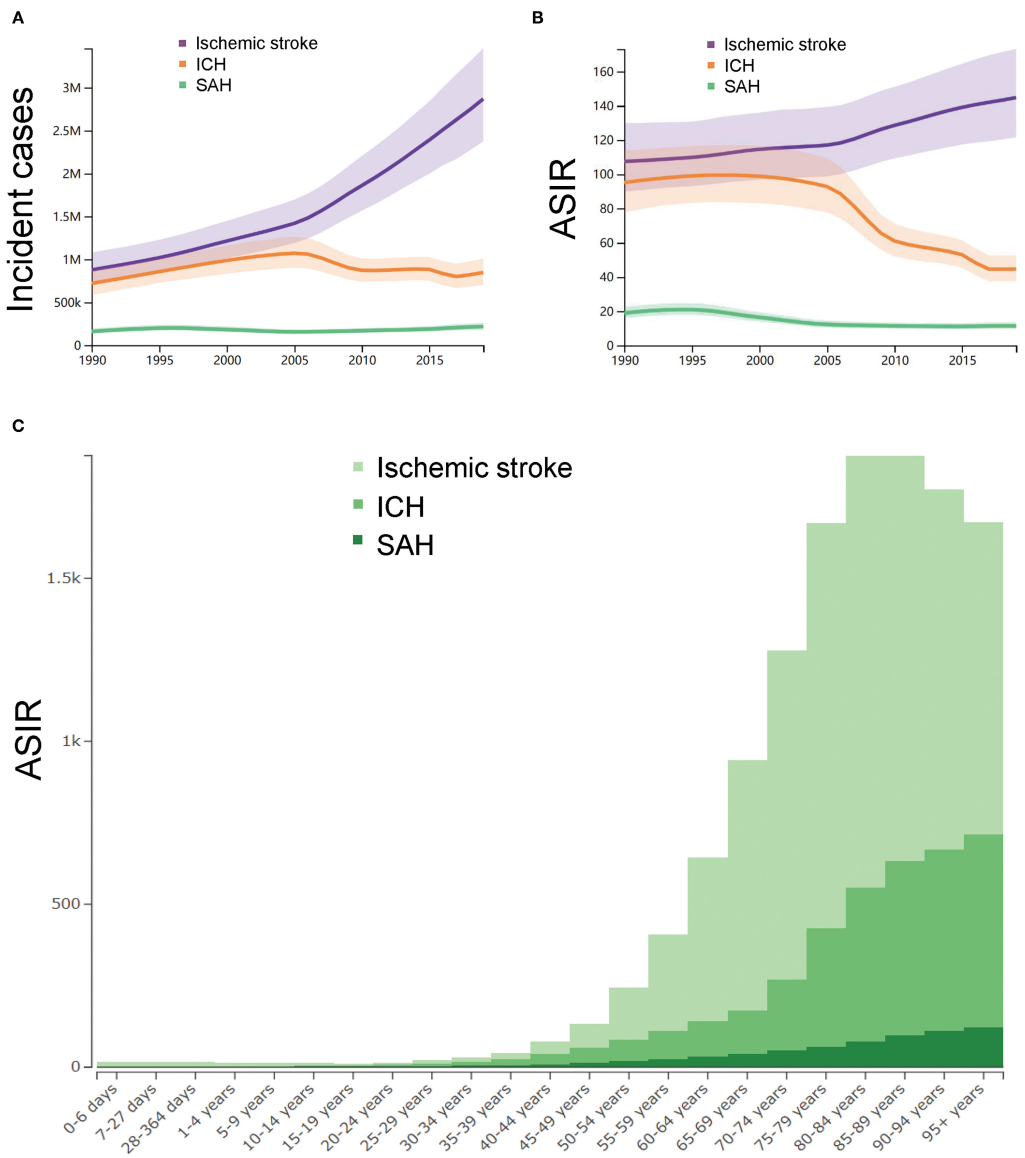
The trends in incidence of strokes. **(A)** Number of the incident cases with uncertainty from 1990 to 2019 in China. **(B)** Age-standardized incidence rate per 100,000 with uncertainty from 1990 to 2019 in China. **(C)** The age-standardized incidence rates per 100,000 of different age groups. ASIR, age-standardized incidence rate; ICH, intracerebral hemorrhage; and SAH, subarachnoid hemorrhage. The figures were generated through GBD Results Tool and GBD Compare Tool.

Supplementary Table 1 showed the summary of the incidence of stroke in China from 1990 to 2019. In 1990, as shown in Figure 1A, the number of incident cases of stroke in China was 1.7 (95% *UI* 1.5–2.0) million, of whom 0.8 (0.7–1.0) million were ischemic stroke, 0.7 (0.5–0.8) million were ICH, and 0.1 (0.1–0.2) million were SAH. As shown in Figure 1B, the ASIR (per 100,000) of stroke in China was 221 (196–249) in 1990 (ischemic stroke: 107, 95% *UI* 89–130; ICH: 95, 95% *UI* 77–113; SAH: 18, 95% *UI* 15–22). In 2019, the number of incident cases of stroke in China was 3.9 (95% *UI* 3.4–4.5) million, of whom 2.8 (2.3–3.4) million were ischemic stroke, 0.8 (0.7–1.0) million were ICH, and 0.2 (0.1–0.2) million were SAH. The ASIR (per 100,000) of stroke in China was 200 (176–230) in 2019 (ischemic stroke: 144, 95% *UI* 121–173; ICH: 44, 95% *UI* 37–52; SAH: 11, 95% *UI* 9–13). From 1990 to 2019, the number of incident cases of stroke in China were increased by 124.0% (108.0–129.0) between 1990 and 2019. The number of incident cases of ischemic stroke, ICH, and SAH in China were increased by 226.0% (211.0–243.0), 18.0% (12.0–24.0), and 36.0% (26.0–45.0), respectively. The ASIR (per 100,000) of stroke in China were decreased by −9.0% (−16.0 to −3.0) between 1990 and 2019. Specifically, the ASIR of ischemic stroke were increased by 35.0% (29.0–40.0) while the ASIR of ICH and SAH were decreased by −53.0% (−56.0 to −50.0) and by −39.0% (−44.0 to −35.0), respectively.

The ASIR (per 100,000) of ischemic stroke, ICH and SAH in 2019 across different age groups were synchronously analyzed, as shown in Figure 1C. The subgroup aged 80–84 years (1326, 95% *UI* 944–1801), 85–89 years (1242, 95% *UI* 953–1588), and 75–79 years 1242, 95% *UI* 893–1664) had the highest ASIR of ischemic stroke. The subgroup aged 95 years or older (591, 95% *UI* 329–1007), 90–95 years (555, 95% *UI* 370–865), and 85–89 years (534, 95% *UI* 398–732) had the highest ASIR of ICH. The subgroup 95 years or older (122, 95% *UI* 77–179), 90–95 years (111, 95% *UI* 80–150), and 85–89 years (97, 95% *UI* 75–124) had the highest ASIR of SAH.

**Figure 2:**
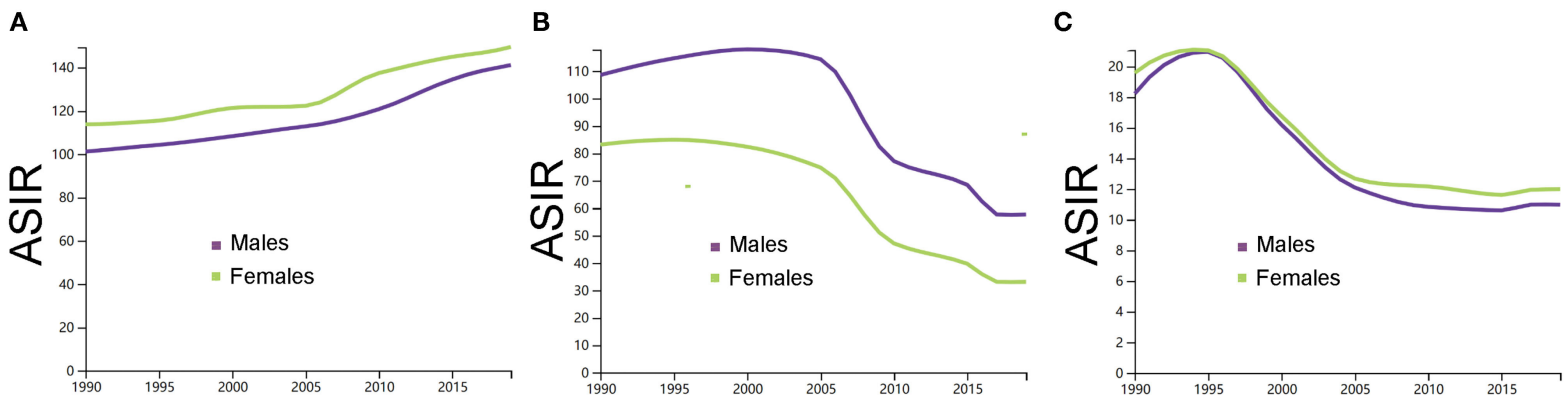
**(A)** Comparison of men and women in the age-standardized incidence rate per 100,000 of ischemic stroke. **(B)** Comparison of men and women in the age-standardized incidence rate per 100,000 of intracerebral hemorrhage. **(C)** Comparison of men and women in the age-standardized incidence rate per 100,000 of subarachnoid hemorrhage. ASIR, age-standardized incidence rate; ICH, intracerebral hemorrhage; and SAH, subarachnoid hemorrhage. The figures were generated through GBD Results Tool.

Figure 2 showed the trends in the incidence rates of ischemic stroke, ICH, and SAH across the different sexes between 1990 and 2019. In 1990, the ASIR of ischemic stroke was 101 (84–121) for men and 113 (94–137) for women, the ASIR of ICH was 108 (88–130) for men and 83 (67–100) for women, and the ASIR of SAH was 18 (15–21) for men and 19 (16–23) for women. In 2019, the ASIR of ischemic stroke was 141 (118–168) for men and 149 (124–179) for women, the ASIR of ICH was 57 (48–67) for men and 33 (27–39) for women, and the ASIR of SAH was 10 (9–12) for men and 11 (10–14) for women.

### Mortality

**Figure 3:**
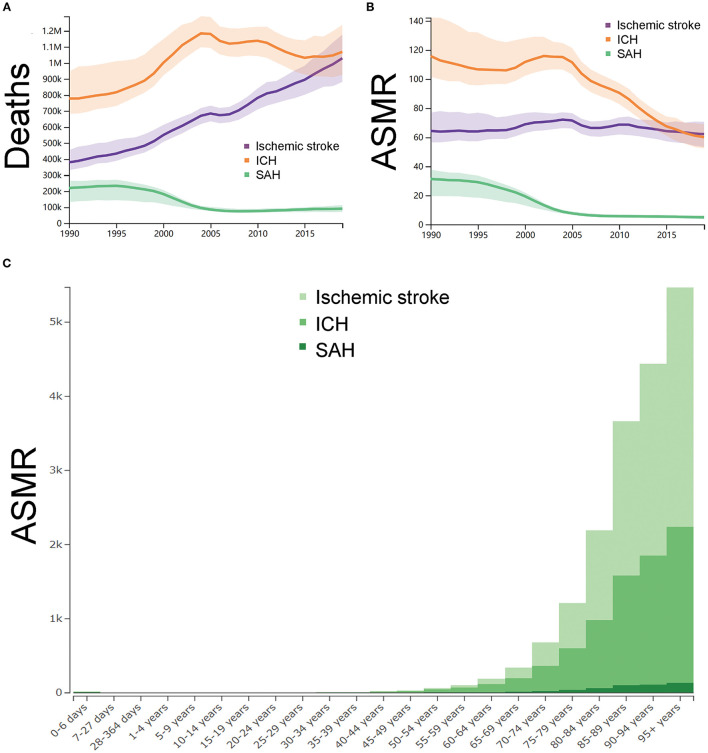
The trends in mortality of strokes. **(A)** Number of deaths with uncertainty from 1990 to 2019 in China. **(B)** Age-standardized mortality rate per 100,000 with uncertainty from 1990 to 2019 in China. **(C)** The age-standardized mortality rates per 100,000 of different age groups. ASMR, age-standardized mortality rate ICH, intracerebral hemorrhage; and SAH, subarachnoid hemorrhage. The figures were generated through GBD Results Tool and GBD Compare Tool.

Supplementary Table 1 showed the summary of the mortality of stroke in China from 1990 to 2019. In 1990, as shown in Figure 3A, the number of deaths of stroke in China was 1.3 (95% *UI* 1.2–1.5) million, of whom 0.3 (0.3–0.4) million were ischemic stroke, 0.7 (0.6–0.9) million were ICH, and 0.2 (0.1–0.2) million were SAH. As shown in Figure 3B, the ASMR (per 100,000) of stroke in China was 211 (187–243) in China in 1990 (ischemic stroke: 64, 95% *UI* 56–76; ICH: 115, 95% *UI* 101–142; SAH: 31, 95% *UI* 19–37). In 2019, the number of deaths of stroke in China was 2.1 (95% *UI* 3.4–4.5) million, of whom 1.0 (0.8–1.1) million were ischemic stroke, 1.0 (0.9–1.2) million were ICH, and 0.1 (0.07–0.11) million were SAH. The ASMR (per 100,000) of stroke in China was 127 (110–144) in 2019 (ischemic stroke: 62, 95% *UI* 53–70; ICH: 60, 95% *UI* 52–68; SAH: 5, 95% *UI* 3–6). From 1990 to 2019, the number of deaths of stroke in China were increased by 59.0% (31.0–91.0). The number of deaths due to ischemic stroke and ICH in China were increased by 171.0% (109.0–228.0) and 37.0% (10.0–66.0) while the number of deaths due to SAH were decreased by −59.0% (−71.0 to −19.0). The ASMR (per 100,000) of stroke in China were decreased by −40.0% (−51.0 to −29.0) between 1990 and 2019. Specifically, the ASMR of ischemic stroke were increased by 3.0% (−26.0 to 16.0) while the ASMR of ICH and SAH were decreased by −48.0% (−59.0 to −38.0) and by −84.0% (−89.0 to −69.0), respectively.

The ASMR (per 100,000) of ischemic stroke, ICH and SAH in 2019 across the different age groups were synchronously analyzed. As shown in Figure 3C, the subgroup aged 95 years or older (ischemic stroke: 3,226, 95% *UI* 2,531–3,770; ICH: 2,107, 95% *UI* 1668–2460; SAH: 136, 95% *UI* 100–171), 90–95 years (ischemic stroke: 2,583, 95% *UI* 2111–2,989; ICH: 1,741, 95% *UI* 1,447–2,019; SAH: 112, 95% *UI* 85–143), and 85–89 years (ischemic stroke: 2080, 95% *UI* 1788–2352; ICH: 1,478, 95% *UI* 1275–1672; SAH: 101, 95% *UI* 80–129) had the highest ASMR.

**Figure 4:**
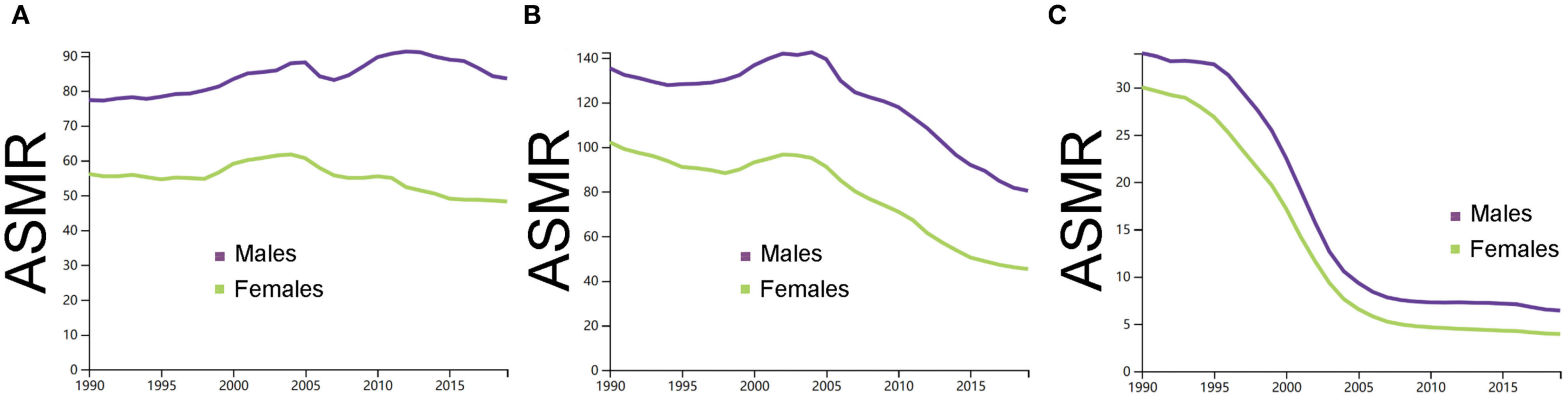
**(A)** Comparison of men and women in the age-standardized mortality rate per 100,000 of ischemic stroke. **(B)** Comparison of men and women in the age-standardized mortality rate per 100,000 of intracerebral hemorrhage. **(C)** Comparison of men and women in the age-standardized mortality rate per 100,000 of subarachnoid hemorrhage. ASMR, age-standardized mortality rate; ICH, intracerebral hemorrhage; and SAH, subarachnoid hemorrhage. The figures were generated through GBD Results Tool.

Figure 4 showed the trends in mortality rate of ischemic stroke, ICH and SAH across the different sexes between 1990 and 2019. In 1990, the ASMR of ischemic stroke was 77 (66–95) for men and 56 (47–67) for women, the ASMR of ICH was 135 (114–171) for men and 102 (86–123) for women, and the ASMR of SAH was 33 (10–44) for men and 30 (23–35) for women. In 2019, the ASMR of ischemic stroke was 83 (69–97) for men and 48 (38–58) for women, the ASMR of ICH was 80 (66–95) for men and 45 (36–54) for women, and the ASMR of SAH was 6 (4–8) for men and 3 (3–4) for women.

## Discussion

The GBD, injuries, and risk factors study is the unique and systematic assessment of the global burden of 354 diseases and injuries by age, sex, and location, and is updated annually (10, 11, 14). Although the previous studies have reported the global burden of stroke based on the GBD study 2016, the separate estimates for stroke in China are lacking (4, 5). Our study is the first in providing the trends in the incidence and mortality of strokes, such as ischemic stroke, ICH, and SAH in China based on the latest GBD study 2019. Although the number of incident cases and deaths nearly doubled from 1990 to 2019, the overall incidence rates and mortality rates of stroke in China have decreased. The reduction in the incidence rates and mortality rates were possibly ascribed to the sophisticated diagnosis, improved treatments, and the implement of multidisciplinary therapy, such as neurosurgical care and neurocritical care. The population growth and aging in China are likely contributed to the increase in the number of incident cases and deaths (15).

Although the incidence rates of ICH and SAH decrease from 1990 to 2019, the increase in the incidence rates of ischemic stroke are observed. Moreover, it appears the mortality rates of ischemic stroke were nearly similar between 1990 and 2019. Early detection and diagnosis for ischemic stroke are likely attributed to the increase in the incidence rates.

Based on our analysis, a growing burden due to ischemic stroke in China are observed. The results are also accordant with the findings in the United States, which indicate that ischemic stroke, accounting for 87% of the total strokes with an increasing trend, have currently become a major problem and the main focus of stroke research in the United States (16). In these contexts, our study provides crucial information to implement the strategies on the first-class prevention, early detection, prehospital management, and multidisciplinary approach.

As refers to the sex-specific differences, little is known about the sex differences in the incidence and mortality of stroke in China. It has been long recognized that the incidence rate of stroke is higher in the men than in women (17, 18). But our study suggests the ischemic stroke incidence is higher in women in China in the past 30 years, and the SAH incidence is also higher in women from 2000 compared to the men. Additionally, although the International Stroke Outcomes Study conducted in Australia, the Caribbean, Europe, and South America suggests women have higher mortality compared with the men (19), a higher mortality rate in men is seen in our study. One possible explanation to the discrepancy between our study and some previous study is the differences in the social and behavior contexts (20).

Our study also indicates the burden of stroke in China is relatively high compared with some high-income and developed countries, such as Australia and the United States (14, 21, 22). The stroke burden attributable to the risk factors in China should be further analyzed.

### Limitations

The current work has some limitations. First, the largest limitation of our study is the availability of primary data. GBD study is lack of high-quality epidemiological data for some low-middle income countries. Second, this study is lack of the estimates for the different provinces. However, we believe the systematic analysis of the burden of stroke in China should be the foundation before more detailed data are analyzed, which will be addressed in the further study. Our study did not analyze the attributable to the risk factors due to stroke, which will be further analyzed.

## Data Availability Statement

Publicly available datasets were analyzed in this study. This data can be found here: The Institute for Health Metrics and Evaluation (IHME) Global Health Data Exchange (GHDx), http://ghdx.healthdata.org/gbd-results-tool.

## Author Contributions

SC, XZ, and TS: conceptualization. KW and CY: formal analysis. SC and TS: methodology and writing—original draft. XZ: investigation. KW: software. SC and CY: funding acquisition. XZ, MS, and CY: writing—review and editing. All authors have agreed to be listed and have seen and approved the manuscript.

## Funding

This study was supported by the West China Fourth Hospital, Sichuan University (No. HLB0021) and 1.3.5 project for disciplines of excellence of the West China Hospital, Sichuan University (No. 2018HXFH010).

## Conflict of Interest

The authors declare that the research was conducted in the absence of any commercial or financial relationships that could be construed as a potential conflict of interest.

## Publisher's Note

All claims expressed in this article are solely those of the authors and do not necessarily represent those of their affiliated organizations, or those of the publisher, the editors and the reviewers. Any product that may be evaluated in this article, or claim that may be made by its manufacturer, is not guaranteed or endorsed by the publisher.
