# Supplementary material for: Trends in Incidence and Mortality of Stroke in China From 1990 to 2019
Source: Front Neurol. 2021 Nov 22;12:759221. doi: 10.3389/fneur.2021.759221 (PMC8645546; doi:10.3389/fneur.2021.759221)
Supplement: Supplementary file 1 [file Table_1.DOCX]

**Supplementary Table 1** Summary of the incidence and mortality of stroke in China from 1990 to 2019

|  | Incidence | | | Mortality | |  |
| --- | --- | --- | --- | --- | --- | --- |
|  | Numbers  (95% UI) | Rate  (95% UI) | Numbers  (95% UI) | | Rate  (95% UI) | |
| 1990 |  |  |  | |  | |
| Females | 0.8 m  (0.7-0.9) | 216.4  (192.1-245.1) | 0.6 m  (0.5-0.7) | | 188.2  (161.7-224.2) | |
| Males | 0.8 m  (0.7-1.0) | 227.9  (202.8-257.3) | 0.7 m  (0.5-0.8) | | 246.3  (212.6-286.8) | |
| Both | 1.7 m  (1.5-2.0) | 221.5  (196.8-249.6) | 1.3 m  (1.2-1.5) | | 211.4  (187.6-243.8) | |
| 2000 |  |  |  | |  | |
| Females | 1.1 m  (1.0-1.3) | 220.4  (197.1-249.2) | 0.7 m  (0.7-0.8) | | 169.3  (151.0-191.8) | |
| Males | 1.2 m  (1.0-1.3) | 242.3  (216.9-271.8) | 0.9 m  (0.8-1.0) | | 242.5  (222.6-268.3) | |
| Both | 2.3 m  (2.1-2.6) | 229.8  (205.6-258.6) | 1.7 m  (1.6-1.9) | | 200.0  (184.4-221.5) | |
| 2010 |  |  |  | |  | |
| Females | 1.4 m  (1.2-1.6) | 196.7  (175.4-223.9) | 0.8 m  (0.7-0.9) | | 214.8  (195.0-243.1) | |
| Males | 1.4 m  (1.2-1.6) | 208.7  (188.0-234.0) | 1.1 m  (1.0-1.2) | | 131.1  (116.9-144.2) | |
| Both | 2.9 m  (2.5-3.2) | 201.0  (180.1-226.4) | 1.9 m  (1.8-2.1) | | 164.9  (150.2-176.8) | |
| 2019 |  |  |  | |  | |
| Females | 1.9 m  (1.7-2.3) | 194.5  (169.6-225.1) | 0.9 m  (0.7-1.1) | | 97.4  (78.8-117.0) | |
| Males | 1.9 m  (1.7-2.2) | 209.7  (185.8-239.3) | 1.2 m  (1.0-1.5) | | 170.3  (141.8-200.1) | |
| Both | 3.9 m  (3.4-4.5) | 200.8  (176.9-230.8) | 2.1 m  (1.8-2.5) | | 127.2  (110.2-144.8) | |

UI, uncertainty intervals; m, million.
